# Isoflurane increases the activity of the vascular matrix metalloproteinase-2 in non-pregnant rats and increases the nitric oxide metabolites in pregnancy

**DOI:** 10.1042/BSR20240192

**Published:** 2024-05-31

**Authors:** Carolina Rosa Rodrigues Souza, Edileia Souza Paula Caetano, Serginara David Rodrigues, Matheus Cleto Lopes, Bruna Rahal Mattos, Mariana Landenberger Santos, Elen Rizzi, Carlos A. Dias-Junior

**Affiliations:** 1 Department of Biophysics and Pharmacology, Institute of Biosciences of Botucatu, Sao Paulo State University (UNESP), Botucatu, Sao Paulo, Brazil; 2 School of Veterinary Medicine and Animal Science, UNESP, Botucatu, Sao Paulo, Brazil; 3 Unit of Biotechnology, University of Ribeirao Preto, UNAERP, Ribeirao Preto, Sao Paulo, Brazil

**Keywords:** isoflurane, metalloproteinase, nitric oxide, non-pregnant rats, pregnancy

## Abstract

Surgeries that require general anesthesia occur in 1.5–2% of gestations. Isoflurane is frequently used because of its lower possibility of affecting fetal growth. Therefore, we examined the isoflurane anesthesia-induced effects on maternal hemodynamic and vascular changes. We hypothesized that isoflurane would enhance endothelium-dependent vasodilation as a consequence of increased nitric oxide and decreased metalloproteinases (MMPs).

Female rats (*n*=28) were randomized into 4 groups (7 rats/group): conscious (non-anesthetized) non-pregnant group, non-pregnant anesthetized group, conscious pregnant group, and pregnant anesthetized group. Anesthesia was performed on the 20th pregnancy day, and hemodynamic parameters were monitored. Nitric oxide metabolites, gelatinolytic activity of MMP-2 and MMP-9, and the vascular function were assessed.

Isoflurane caused no significant hemodynamic changes in pregnant compared with non-pregnant anesthetized group. Impaired acetylcholine-induced relaxations were observed only in conscious non-pregnant group (by approximately 62%) versus 81% for other groups. Phenylephrine-induced contractions were greater in endothelium-removed aorta segments of both pregnant groups (with or without isoflurane) compared with non-pregnant groups. Higher nitric oxide metabolites were observed in anesthetized pregnant in comparison with the other groups. Reductions in the 75 kDa activity and concomitant increases in 64 kDa MMP-2 isoforms were observed in aortas of pregnant anesthetized (or not) groups compared with conscious non-pregnant group.

Isoflurane anesthesia shows stable effects on hemodynamic parameters and normal MMP-2 activation in pregnancy. Furthermore, there were increases in nitric oxide bioavailability, suggesting that isoflurane provides protective actions to the endothelium in pregnancy.

## Introduction

General anesthesia is usually the option choice during urgent and emergent non-obstetric and obstetric surgeries in pregnant women, once neuraxial/regional anesthesia may have maternal contraindication [1,2]. Although few studies had evaluated the influence of the inhalation of general anesthetics in the vascular and hemodynamics changes during pregnancy [3–6], the risks and recommendation of general anesthesia in pregnancy remain unclear.

Among general inhalational anesthetics, isoflurane is routinely used [1]. Isoflurane has low solubility in blood and tissues, which results in rapid recovery of the mother and fetus [7,8], being less likely to cause fetal growth restriction. However, studies have been shown that isoflurane may induce cardiovascular changes including decreased cardiac contractility, altered arterial compliance [9] and interference with the vasomotor response [10]. Moreover, it has also been found that isoflurane induced nitric oxide formation [11,12], but the isoflurane anesthesia-induced effects are still unclear in pregnancy.

It has been suggested that nitric oxide negatively modulates the activity of matrix metalloproteinases (MMPs) because reductions in nitric oxide levels have been correlated with increases in activities of MMP-2 and MMP-9 [13,14] including during late pregnancy [15,16]. Pregnancy-associated vascular remodeling is regulated, at least in part, by gelatinolytic activity of MMPs [17]. Moreover, the excess and exacerbation of the activity of these proteases may trigger atherosclerosis, aneurysm, and other cardiovascular diseases [18]. However, whether pregnancy-associated changes in circulating levels of nitric oxide and activity of MMPs of the maternal vasculature would be affected by isoflurane anesthesia are not clearly understood.

Therefore, we examined hemodynamic and vascular function changes in non-pregnant group and pregnant rats anesthetized with isoflurane at the final stage of pregnancy. We hypothesized that anesthesia with isoflurane would enhance endothelium-dependent vasodilation, and that this effect would be related to the increases in nitric oxide levels and decreases in activity of MMP-2 and MMP-9. This hypothesis is supported by previous studies showing that isoflurane caused increase in nitric oxide production [11,12].

## Methods

### Experimental design and animal protocol

Wistar rats (200–250 g) were obtained from the Central Vivarium of the São Paulo State University (UNESP - Botucatu Campus) and were kept in the Department of Pharmacology, and were housed in cages, on a 12-h light/dark cycle, under controlled temperatures (23 ± 2°C) with free access to rat chow and water [19]. Female rats were mated overnight and the first day of pregnancy was defined when spermatozoa were found in a vaginal smear containing anucleated cornified squamous cells.

Timed-female rats (*n*=28), 12 weeks old, were equally randomized into four groups (7 rats/group): conscious (non-anesthetized) non-pregnant rats (Non-Preg group), non-pregnant rats anesthetized with isoflurane (Non-Preg+Iso group), conscious (non-anesthetized) pregnant rats (Preg group), and pregnant rats anesthetized with isoflurane (Preg+Iso group). Additionally, anesthesia was performed on the 20th day of pregnancy.

The isoflurane anesthesia was induced with a 3% concentration in a glass chamber connected to the anesthesia system for rodents (AI, Insight, Ribeirao Preto, Brazil). After induction, the animals were kept in dorsal decubitus, with inhalation anesthetic (isoflurane in concentration range between 1.5–2% and oxygen 100%) administered through a face mask, by spontaneous ventilation, and maintained under these conditions for 150 min. The isoflurane concentrations were recorded by the anesthetic gas analyzer (Vamos Plus, Dräger, Lübeck, Germany).

### Evaluation of hemodynamic parameters and euthanasia

Systolic, diastolic, and mean arterial pressure, and heart rate (SAP, DAP, MAP, and HR respectively) were measured using a tail‐cuff plethysmography (Insight, Ribeirao Preto, Sao Paulo, Brazil, catalog #EFF 306) at 30, 45, 60, 75, 90, 105, 120, 135, and 150 min (T_30_, T_45_, T_60_, T_75_, T_90_, T_105_, T_120_, T_135_, and T_150_ time points, respectively) after isoflurane induction in the anesthetized rats (Non-Preg+Iso and Preg+Iso groups) and before the euthanasia in the conscious (non-anesthetized) rats (Non-Preg and Preg groups).

Body temperature was measured using a rectal temperature probe connected to a multi-parameter monitor (DX monitor, Dixtal Biomedica, São Paulo, Brazil).

Euthanasia was performed by decapitation and exsanguination with no anesthesia in rats from the Non-Preg and Preg groups, and by cardiac puncture (with a needle inserted into the left ventricle) in the anesthetized rats from the Non-Preg+Iso and Preg+Iso groups.

Abdominal and thoracic aortas were collected after the procedure. Abdominal aorta segments were used in vascular reactivity experiments, and thoracic aortas fragments were stored at −80°C for further biochemical analyzes.

Heparinized blood obtained right after euthanasia was centrifuged at 10.000 rpm (for 10 min), and plasma was separated and stored at --20°C.

The number and weight of fetuses and placentae of each pregnant rat were recorded.

### Isometric contraction and relaxation

**Figure 1:**
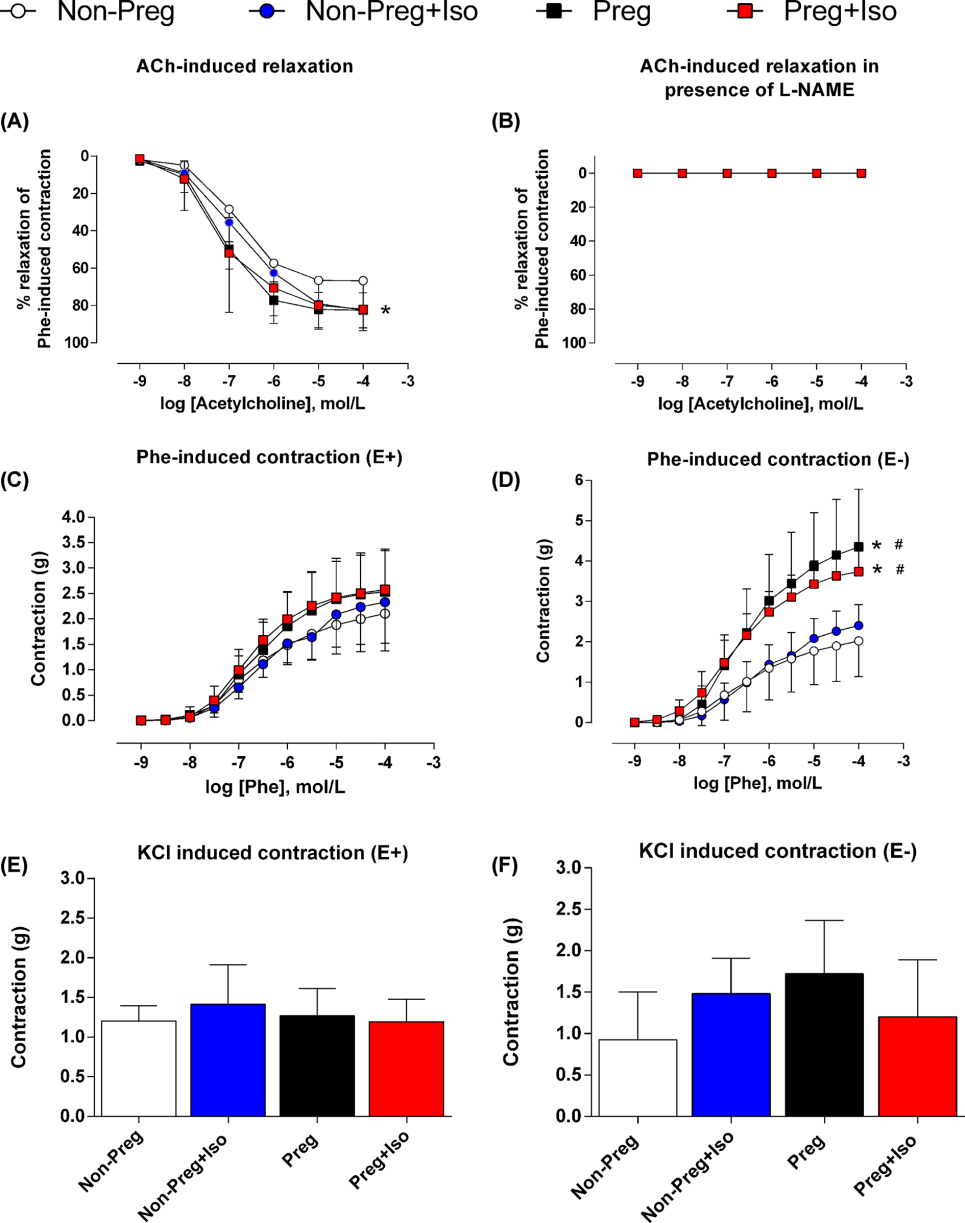
Isometric contraction and relaxation of the abdominal aorta (**A**) Acetylcholine (ACh)-induced vasodilation, (**B**) ACh-induced vasodilation in the presence of L-NAME, (**C**) Phenylephrine (Phe)-induced contraction in endothelium-intact aorta rings, (**D**) Phe-induced contraction in endothelium-denuded aorta rings, (**E**) KCl induced contraction in endothelium-intact aorta rings, (**F**) KCl induced contraction in endothelium-denuded aorta rings were evaluated in abdominal aorta rings of Non-Preg, Non-Preg+Iso, Preg, and Preg+Iso groups. Values represent mean ± SD. *P<0.05 versus Non-Preg, #*P*<0.05 versus Non-Preg+Iso.

Abdominal aorta was dissected and divided into four segments (3–4 mm in length) as follows: two segments with intact endothelium (E^+^) and two segments in which endothelium was mechanically removed (E^−^). With the aid of a dissection microscope, the endothelium was removed by scraping the vessel interior (five times) around the tip of forceps. Abdominal aorta segments were placed in organ chambers containing 10 ml of Krebs-Henseleit saline solution, bubbled with 95% O_2_ and 5% CO_2_ at 37°C, as previously described [20]. Briefly, all abdominal aorta segments were stretched until an optimal basal tension (1.5 g), and then the segments were allowed to equilibrate for 60 min, and bath physiological solution was changed every 15 min. The basal tensions (*g*) depicted in Figure 1 for phenylephrine is the active tension. The resting tension for all segments was not different.

Changes in isometric tensions were recorded using isometric transducers FORT10 (WPI, U.S.A.) connected to a PC-based MP100 System and analyzed offline using the AcqKnowledge 3.5.7 software (Biopac Systems Inc., Goleta, CA, U.S.A.). After stabilization, the contractile activity of vascular smooth muscle was challenged to maximal (96 mM) potassium chloride (KCl)-induced contraction and cumulative concentrations of phenylephrine (Phe, 10^−9^ to 10^−4^M). To determine endothelial function, abdominal aorta segments were challenged with increasing concentrations of acetylcholine (ACh, 10^−9^ to 10^−4^ M) in pre-contracted rings with Phe (10^−6^ M) [20,21]. To examine the impact of isoflurane anesthesia on endothelium-dependent vasodilation, ACh concentration-response curves were constructed in E^+^ and E^−^ aorta segments pre-contracted with Phe (10^−6^ M), pre-incubated (or not) with a non-selective inhibitor of nitric oxide syntheses: the Nω-nitro-L-arginine-methyl ester (L-NAME, 3 × 10^−4^ M).

### Determination of nitric oxide metabolites (nitrite and nitrate)

Nitrite and nitrate concentrations were evaluated in plasma using Griess reagents, as described previously [22]. Briefly, concentration of nitrite and nitrate (NOx) was calculated using a standard sodium nitrite curve (1.56–100 μM) and the absorbance was read on a spectrophotometer at 535 nm (Synergy 4, BIOTEK, Winooski, VT, U.S.A.). NOx levels in plasma were expressed in μmol/L.

### Determination of the gelatinolytic activity of MMP-2 and MMP-9

This analysis was performed using the gelatin zymography technique in thoracic aorta and plasma, as described previously [23]. Gelatinolytic activities were expressed as arbitrary units. The MMP-2 forms were identified as bands at 75, 72, and 64 kDa, and MMP-9 as band at 92 kDa.

### Data analysis and statistics, and ethical aspects of research

The results are expressed as mean ± SD. Using Graph Pad Prism (Software version 6.0, San Diego, CA, U.S.A.), a Shapiro–Wilk test was applied to verify normality of data distribution. No data was excluded from the analysis in any experiment. The hemodynamic values registered in anesthetized rats (Non-Preg+Iso and Preg+Iso groups) and conscious rats (Non-Preg and Preg groups) were assessed by two-way (isoflurane × time) analysis of variance followed by Tukey’s post*-*hoc test, while fetal and placental weight, and biochemical recordings were assessed by Student’s *t-*test. For vascular reactivity experiments, individual concentration contraction curves were constructed and sigmoidal curves were fitted to the data using the least square method. The values of the maximum effect achieved (*E*_max_) and the negative logarithmic of the concentration producing 50% of maximal effect (pEC_50_) were calculated. To compare *E*_max_ and pEC_50_ values, we used one-way analysis of variance (ANOVA), and the Tukey’s test for post-hoc analysis was applied, while two-way ANOVA was handled to examine the differences among four groups. Value of *P*<0.05 was considered significant.

All procedures for animal experiments were approved by the local Committee for Ethics in Animal Experimentation of the Institute of Biosciences, UNESP, Botucatu, Sao Paulo, Brazil on August 26, 2019 (protocol number: 1190/2019), which were in accordance with the Animal Research: Reporting of *In Vivo* Experiments (ARRIVE) guidelines [24].

## Results

### Effects of isoflurane on hemodynamic parameters of non-pregnant and pregnant rats, and on fetal and placental parameters

No significant differences were observed regarding the systolic, diastolic, mean arterial pressure, and heart rate values between Preg+Iso and Non-Preg+Iso groups (*P*>0.05, Table 1) neither between Non-Preg and Preg groups (*P*>0.05, Table 1). Similarly, the body temperature also showed no significant difference among the groups (*P*>0.05, Table 1). However, significant differences were observed regarding the systolic arterial pressure values between Preg+Iso and Non-Preg groups (*P*<0.05, Table 1).

**Table 1:** Hemodynamic parameters in non-anesthetized rats (Non-Preg and Preg groups) and anesthetized rats with 1.5–2.0% isoflurane (Non-Preg+Iso and Preg+Iso groups)

Parameters	Groups	Time points (min)								
		T_30_	T_45_	T_60_	T_75_	T_90_	T_105_	T_120_	T_135_	T_150_
**SAP (mmHg)**	**Non-Preg**	117 ± 24	119 ± 20	115± 23	122 ± 18	120 ± 16	117 ± 18	118 ± 17	114± 21	117 ± 22
	**Non-Preg+Iso**	90 ± 8	93 ± 12	90 ± 9	86 ± 9	91 ± 11	87 ± 7	93 ± 13	90 ± 6	84 ± 11
	**Preg**	105 ± 16	107 ± 15	103 ± 15	104 ± 18	102 ± 19	106 ± 17	108 ± 20	106 ± 14	104 ± 16
	**Preg+Iso**	77 ± 7^*^	83 ± 5^*^	87 ± 3^*^	89 ± 2^*^	90 ± 5^*^	91 ± 7^*^	89 ± 10^*^	89 ± 8^*^	89 ± 9^*^
**DAP (mmHg)**	**Non-Preg**	89 ± 22	88 ± 20	86 ± 16	88 ± 19	87 ± 21	88 ± 21	86 ± 22	85 ± 17	89 ± 21
	**Non-Preg+Iso**	59 ± 10	61 ± 9	63 ± 9	60 ± 8	63 ± 7	62 ± 7	69 ± 9	64 ± 6	60 ± 9
	**Preg**	76 ± 15	77 ± 19	75 ± 21	74 ± 22	78 ± 23	76 ± 18	79 ± 17	74 ± 22	76 ± 19
	**Preg+Iso**	55 ± 9	60 ± 8	64 ± 5	65 ± 6	68 ± 9	68 ±11	66 ±11	66 ± 9	66 ± 9
**MAP (mmHg)**	**Non-Preg**	98 ± 23	97 ± 20	99 ± 21	96 ± 22	98 ± 20	97 ± 19	96 ± 21	99 ± 22	96 ± 23
	**Non-Preg+Iso**	69 ± 9	71 ± 8	72 ± 8	69 ± 8	72 ± 7	71 ± 6	77 ± 10	73 ± 6	68 ± 10
	**Preg**	86 ± 15	88 ± 16	85 ± 19	87 ± 18	85 ± 17	88 ± 18	84 ± 17	87 ± 19	85 ± 17
	**Preg+Iso**	62 ± 8	68 ± 7	71 ± 4	73 ± 5	76 ± 7	76 ± 9	73 ±10	74 ± 9	74 ± 9
**HR (bpm)**	**Non-Preg**	330 ± 22	327 ± 19	329 ± 17	326 ± 18	332 ± 20	328 ± 23	329 ± 19	327 ± 20	325 ± 21
	**Non-Preg+Iso**	329 ± 21	326 ± 20	327 ± 24	329 ± 15	329 ± 21	330 ± 19	328 ± 23	330 ± 24	327 ± 20
	**Preg**	328 ± 17	325 ± 18	331 ± 19	327 ± 25	330 ± 18	326 ± 18	325 ± 17	328 ± 21	329 ± 24
	**Preg+Iso**	331 ± 20	322 ± 23	328 ± 21	325 ± 17	328 ± 19	329 ± 21	327 ± 25	326 ± 23	328 ± 19
**Body temperature (°C)**	**Non-Preg**	37.5±0.4	37.6± 0.5	38.3±0.6	38.4±0.7	38.7±0.6	38.3±0.5	38.5±0.7	38.6±0.5	38.3±0.7
	**Non-Preg+Iso**	37.6±0.7	37.9± 0.5	38.1±0.5	38.2±0.4	38.3±0.4	38.3±0.4	38.3±0.5	38.4±0.5	38.4±0.5
	**Preg**	37.7±0.4	38.1± 0.5	37.6±0.7	38.3±0.6	38.5±0.7	38.6±0.5	38.2±0.7	38.5±0.5	38.6±0.4
	**Preg+Iso**	37.5±0.3	37.9± 0.4	38.2±0.5	38.5±0.5	38.8±0.6	38.9±0.7	38.6±0.6	38.7±0.4	38.8±0.5

Data were recorded at 30 (T30), 45 (T45), 60 (T60), 75 (T75), 90 (T90), 105 (T105), 120 (T120), 135 (T135), and 150 min (T150). DAP, diastolic arterial pressure; HR, heart rate; MAP, mean arterial pressure; SAP, systolic arterial pressure. Data represents mean ± SD. Significant differences were derived from two-way analysis of variance followed by Tukey’s post-hoc test.

* *P*<0.05 versus Non-Preg.

After euthanasia, fetal and placental parameters were recorded. Increases in fetal weight were observed in Preg+Iso compared with Preg group (2.83 ± 0.4 g and 2.15 ± 0.4 g respectively; *P=0.001). However, Preg and Preg+Iso groups showed no significant differences in litter size (12.5 ± 2.7 and 10 ± 3.0, respectively; *P*>0.05) and placental weights (0.570 ± 0.2 g and 0.529 ± 0.02 g, respectively; *P*>0.05), which demonstrated similar pregnancy conditions in both pregnant groups.

### Vascular function in abdominal aorta of non-pregnant and pregnant rats anesthetized with isoflurane

Acetylcholine-induced relaxations were higher in Preg+Iso, Preg and Non-Preg+Iso groups compared with Non-Preg group (*P=0.0082, Figure 1A). In addition, no relaxations were observed in any group after L-NAME pre-incubation (*P*>0.05, Figure 1B).

Endothelium-intact aorta showed no significant differences on phenylephrine -induced contractions among the four groups (*P*>0.05, Figure 1C). However, endothelium removal showed differences on phenylephrine-induced contractions, in which both pregnant groups (with or without anesthesia) had higher contractions compared with non-pregnant groups (*^,#^*P*=0.0014, Figure 1D).

Potassium chloride-induced contractions showed no statistical differences among the groups in both endothelium-intact and removed aorta segments (*P*>0.05, Figure 1E,F respectively).

There was no statistical difference in *E*_max_ of phenylephrine-induced contractions in endothelium-intact aorta; however, endothelium-removed aorta showed higher *E*_max_ in both Preg and Preg+Iso groups in comparison with Non-Preg (*P=0.0001 and *P*=0.0042, respectively; Table 2) and Non-Preg+Iso groups (^#^*P*=0.0006 and *P*=0.0409, respectively, Table 2). The pEC_50_ of phenylephrine was higher in Preg+Iso than Non-Preg+Iso group (^#^*P*=0.0149, Table 2).

**Table 2:** Maximal response (*E*_max_) and negative logarithm of the concentration that evoked 50% of the maximal response (pEC_50_) were recorded for phenylephrine (Phe) and acetylcholine (ACh) in abdominal aorta rings, with or without endothelium

	Non-Preg	Non-Preg+Iso	Preg	Preg+Iso
**Phe *E*_max_ (g)**				
**E+**	2.1 ± 0.7	2.3 ± 0.8	2.5 ± 0.8	2.6 ± 0.8
**E-**	2 ± 0.9	2.4 ± 0.5	4.4 ± 1.4^*, #^	3.7 ± 0.7^*, #^
**Phe pEC_50_ (-log M)**				
**E+**	6.7 ± 0.4	6.4 ± 0.7	6.7 ± 0.2	6.8 ± 0.3
**E-**	6.4 ± 0.4	6.2 ± 0.4	6.5 ± 0.3	6.8 ± 0.3^#^
**ACh E_max_ (** **%** **)**				
**E+**	66.6 ± 14.5	82.5 ± 9.5^*^	82.6 ± 9.4^*^	82 ± 11.5^*^
**ACh pEC_50_ (-log M)**				
**E+**	6.8 ± 0.4	6.7 ± 0.7	7.2 ± 0.4	7.1 ± 0.7

Groups: Non-pregnant (Non-Preg), Non- pregnant anesthetized with isoflurane (Non-Preg+Iso), Pregnant (Preg) and Pregnant anesthetized with isoflurane (Preg+Iso) groups. Data represent mean ± SD.

* *P*<0.05 versus Non-Preg group.

# *P*<0.05 versus Non-Preg+Iso group were derived from two-way analysis of variance followed by Tukey’s post-hoc.

The *E*_max_ of acetylcholine was significantly greater in Non-Preg+Iso, Preg and Preg+Iso groups compared with Non-Preg (*P=0.0086, Table 2). In contrast, pEC_50_ values were similar among the groups (*P*>0.05, Table 2).

### Effects of isoflurane on plasma levels of nitric oxide metabolites of non-pregnant and pregnant rats

**Figure 2:**
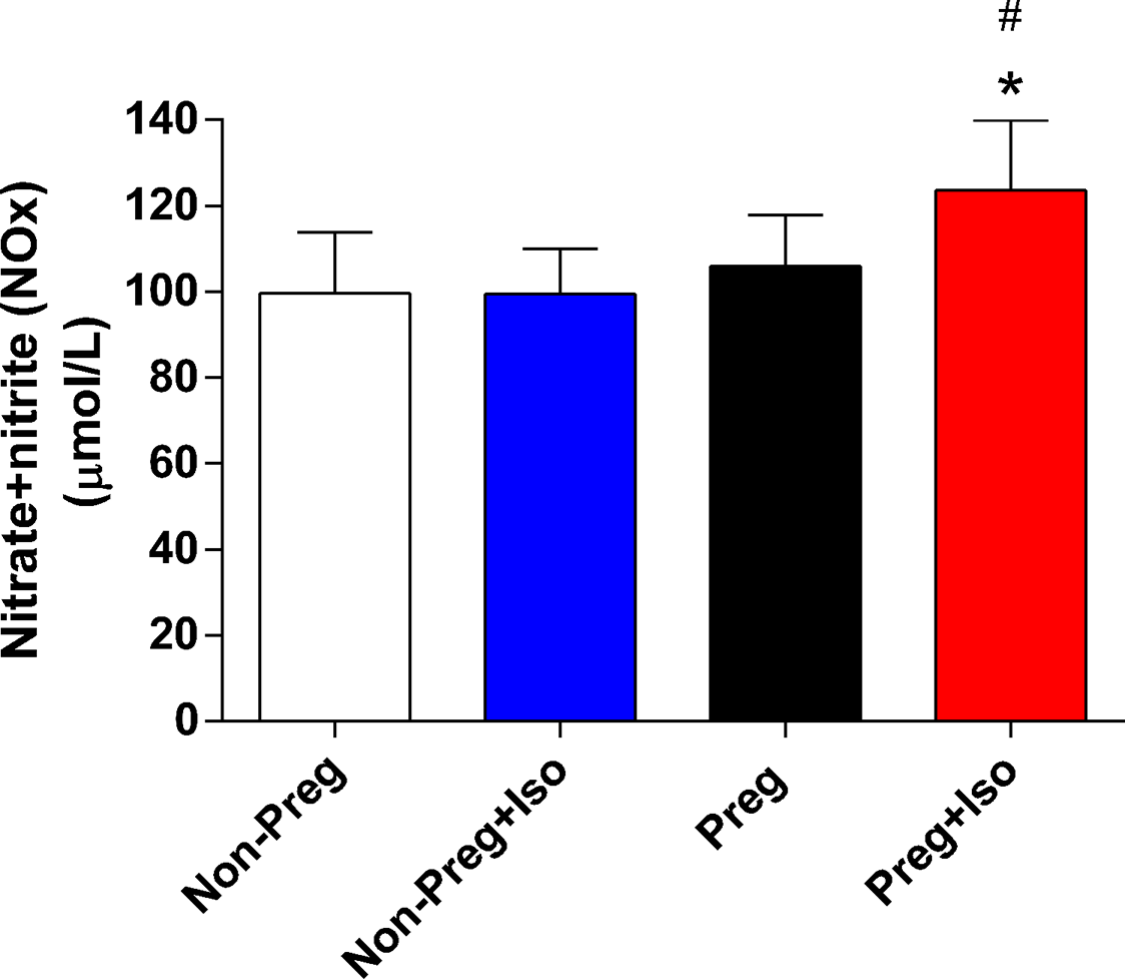
Plasma levels of NOx were determined among the four groups Values represent mean ± SD. *P<0.05 vs Non-Preg, #*P* < 0.05 vs Non-Preg+Iso.

Plasma NOx were significantly higher in Preg+Iso compared with Non-Preg and Non-Preg+Iso groups (*^,#^*P*=0.0096, Figure 2).

### Effects of isoflurane on gelatinolytic activity of MMP-2 in thoracic aorta and MMP-2 and MMP-9 in plasma of non-pregnant and pregnant rats

Zymography technique was performed to determine the gelatinolytic activity of MMP-2 and MMP-9 in aorta fragments. Zymography gels showed no corresponding bands to MMP-9 isoforms, while 75, 72, and 64 kDa MMP-2 isoforms were detected in all groups (Supplementary Figure S1).

**Figure 3:**
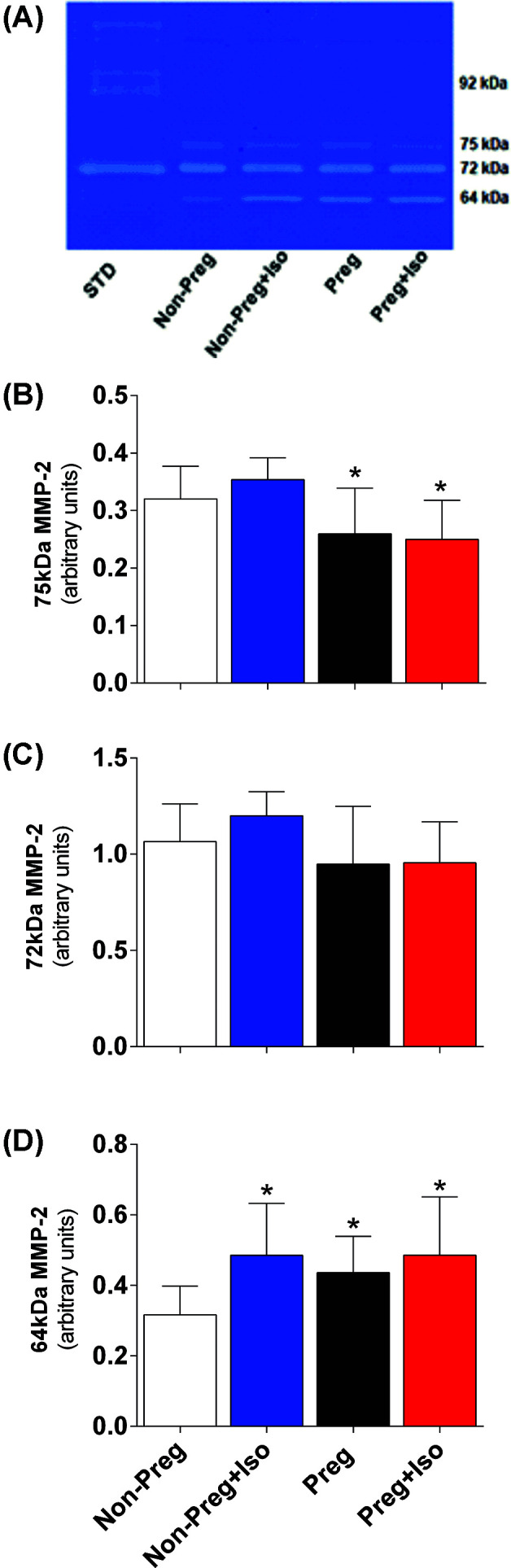
Gelatinolytic activity of MMP-2 in aorta (**A**) Representative zymography gel of aorta, (**B**) gelatinolytic activity of 75 KDa MMP-2, (**C**) 72 KDa MMP-2, and (**D**) 64 KDa MMP-2 examined in thoracic aorta of Non-Preg, Non-Preg+Iso, Preg, and Preg+Iso groups. Values represent mean ± SD. *P<0.05 vs Non-Preg group.

The Preg and Preg+Iso groups showed significantly lower activity of 75 kDa MMP-2 isoform (*P=0.0086, Figure 3B) and a trend of reduction in the 72 kDa MMP-2 isoform (Figure 3C) compared with non-pregnant animals under isoflurane anesthesia or not. However, activity of 64 kDa MMP-2 isoform was statistically higher in Non-Preg+Iso, Preg, Preg+Iso groups compared with Non-Preg group (*P=0.0002, Figure 3D).

Zymography technique was also performed to determine the gelatinolytic activity of MMP-2 and MMP-9 in plasma samples. Zymography gels showed no corresponding bands to 75 and 64 kDa MMP-2 isoforms, while 92 kDa MMP-9 and 72 kDa MMP-2 isoforms were detected in all groups (Supplementary Figure S2).

**Figure 4:**
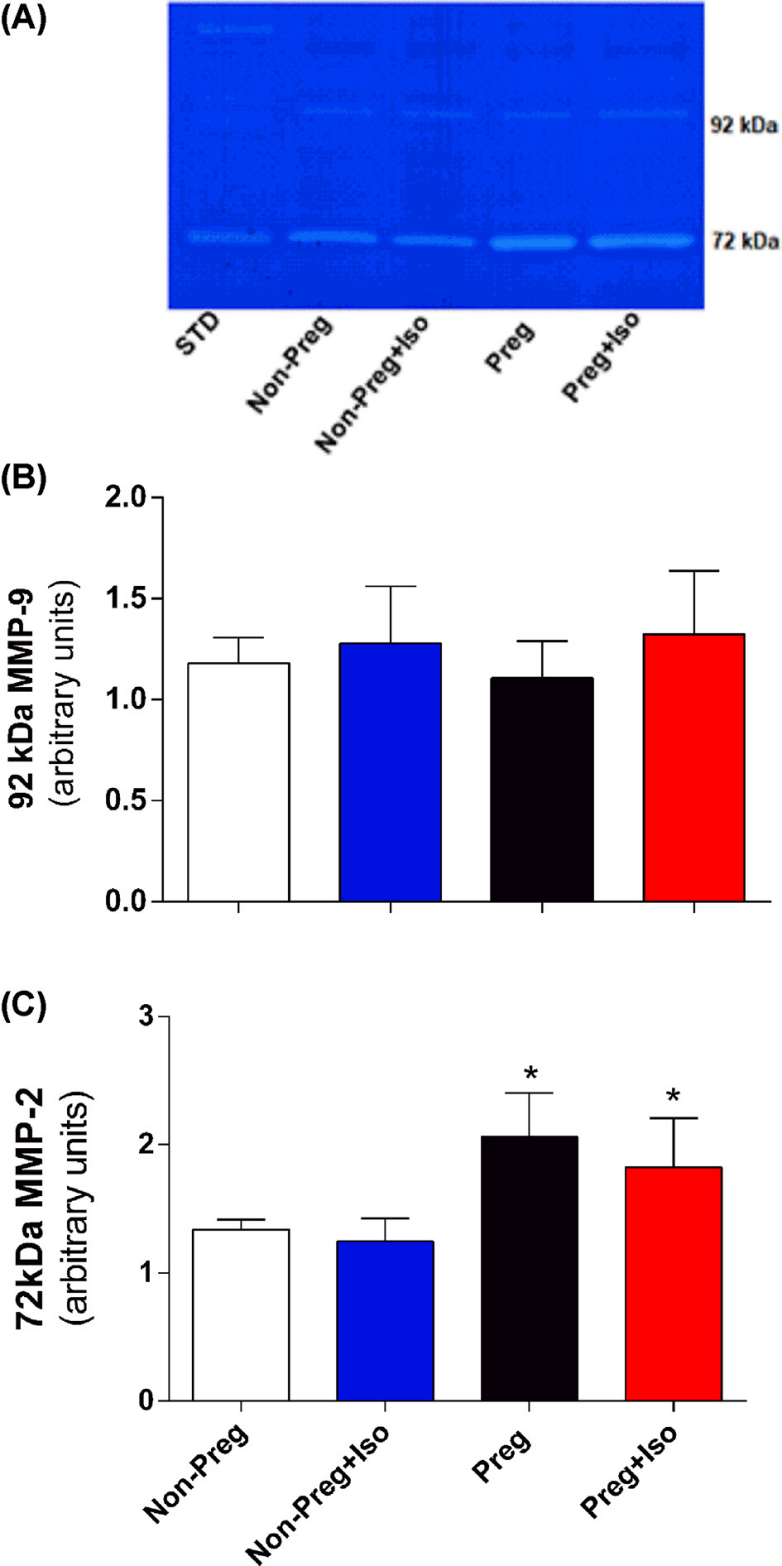
Gelatinolytic activity of MMP-9 and MMP-2 in plasma Representative zymography gel of plasma (**A**), gelatinolytic activity of 92 kDa MMP-9 (**B**) and 72 kDa MMP-2 (**C**) examined in plasma of Non-Preg, Non-Preg+Iso, Preg, and Preg+Iso groups. Values represent mean ± SD. *P<0.05 versus Non-Preg and Non-Preg+Iso groups.

In plasma samples, no differences were found in activity of the 92 kDa MMP-9 isoform among four groups (*P*>0.05, Figure 4B). However, both Preg and Preg+Iso groups showed significantly higher activity of 72 kDa MMP-2 isoform in comparison with Non-Preg and Non-Preg+Iso groups (*P=0.0001, Figure 4C).

## Discussion

This is the first study to examine the isoflurane anesthesia effects on maternal hemodynamic and vascular repercussions at the final stage of pregnancy, linking the nitric oxide bioavailability and the activity of MMPs in pregnancy. The key findings were: firstly, although isoflurane anesthesia showed effect on systolic blood pressure measurements in pregnant rats, vasoconstrictor responses in endothelium-intact abdominal aortas were not affected. Secondly, increases in fetal weights without affecting placental weights and litter size were observed in isoflurane anesthetized group. Thirdly, nitric oxide bioavailability was increased by isoflurane anesthesia in pregnancy, and this effect may be related to the endothelium-derived nitric oxide, as also observed by acetylcholine-induced vasodilation. Lastly, increases in gelatinase activity of MMP-2 associated with vascular physiological alterations induced by pregnancy were not affected by isoflurane anesthesia.

Physiological hemodynamic adaptations occur during pregnancy, including increases in blood volume, cardiac output, and decreases in hematocrit [25]. Importantly, the uterine growing may result in the stasis of venous return from the lower extremity rising the edema events, deep vein thrombosis, intermittently aortocaval compression, and supine hypotension [26]. These alterations may contribute to underlying risks of hypotension and hypoxemia during urgent and emergent surgical procedures that require general anesthesia in pregnancy [27]. Hence, maternal hypotension and hypoxemia should be avoided in order to preserve maternal health and fetal viability under general anesthesia [28]. Notably, the present findings demonstrate that physiological measurements were stable and within normal limits throughout the isoflurane anesthesia. In addition, anesthesia did not cause any effect on vasoconstrictor responses in endothelium-intact aorta, which suggests that the isoflurane is a reliable anesthetic agent, although uterine enlargement is present at the final stage of pregnancy [29].

Uterine blood flow is the most important element of fetal perfusion, and, in physiological conditions, it is preserved in facing the decrease in maternal arterial blood pressure. Such mechanism results in the redistribution of the blood flow to the uterus, thus providing nutrient supply for fetal developing [30]. In our hands, isoflurane anesthesia increased the fetal weights. Furthermore, the present results suggest that isoflurane anesthesia provides more protection in eventual maternal hypotension and hypoxemia, which may be managed by the optimization of the inspired oxygen concentration and by the concomitant administration of vasopressors [28]. Importantly, further investigations during general anesthesia at the final stage of pregnancy are warranted.

Recent findings have indicated that nitric oxide synthase (eNOS)-derived nitric oxide production is triggered by isoflurane [11,12]. Moreover, isoflurane impacts the membrane fluidity and this action may contribute to the changes in eNOS phosphorylation and calcium signaling in endothelial cells, thus achieving far higher nitric oxide production in pregnancy [31]. Furthermore, there is previous evidence that pregnancy up-regulates calcium-activated potassium channel activity in large-conductance arteries and that this pregnancy-associated change can be underlying mechanisms to attenuate contractile tone in uterine arteries [32]. Supporting this previous evidence, our present results revealed that nitric oxide bioavailability and acetylcholine-induced vasodilation are greater in pregnant rats anesthetized with isoflurane. In addition, no significant differences were observed in phenylephrine-induced contractions in endothelium-intact abdominal aorta among the groups, implying that isoflurane anesthesia did not affect vasoconstrictor-induced responses. By contrast, endothelium-removed aorta of both pregnant groups (anesthetized and non-anesthetized) showed greater phenylephrine-induced responses compared with non-pregnant groups, suggesting that isoflurane-induced protective effects observed in pregnancy are endothelium-derived nitric oxide dependent. Moreover, one may consider that the KCl-induced contractions to be as strong as or stronger than the phenylephrine active tension generated. Although our experiments in timed-female rats (12 weeks old) were performed during estrus, to control for endocrine confounders, vascular reactivity can be influenced by sex hormones during estrus cycle, as previously reported [3,21].

The pregnancy-associated adaptations in the blood circulation involve structural remodeling and functional changes in the maternal vasculature [33]. Part of these physiological changes in gestation may be regulated by the MMPs [33]. Pro-MMPs are cleaved into active forms that promote degradation of collagen and other proteins in extracellular matrix [33]. In the present study, gelatin zymography analysis of aorta revealed proteolytic bands corresponding to the pro-MMP-2 isoforms (75 and 72 kDa) and active MMP-2 isoform (64 kDa). We have also examined MMPs in plasma, and gelatin zymography analysis revealed proteolytic bands corresponding to the pro-MMP-9 (92 kDa) and pro-MMP-2 (72 kDa), and that may reflect other maternal tissues that are also sources of MMPs. Our results show no statistical differences among all groups regarding the 72 kDa MMP-2 in the aorta and 92 kDa MMP-9 in the plasma. However, Preg and Preg+Iso groups, in compression with the Non-Preg group, showed decreases in the activity of 75 kDa MMP-2 and increases in the activity of 64 kDa MMP-2 in the aorta, and increases in the activity of 72 kDa MMP-2 in the plasma. The present findings suggest that isoflurane anesthesia has no influence on the MMP-2 activation in pregnancy, and moreover, such mechanism is involved during the remodeling period in healthy gestation since significant hemodynamic, vascular, and uteroplacental adaptations are needed for a proper blood supply during fetal developing [33]. Moreover, increases in the activity of 64 kDa MMP-2 in the Non-Preg+Iso group compared with the Non-Preg group may be explained by pro-oxidant effect of isoflurane anesthesia [34].

Limitations should be considered. Firstly, isoflurane anesthesia in non-pregnant state revealed that gelatinolytic activity of 64 KDa MMP-2 in aorta was greater compared with the respective conscious group, and such alteration must be further investigated. Secondly, our results strongly support that, pregnant women diagnosed with endothelial dysfunction require more attention during urgent and emergent procedures in which isoflurane anesthesia is needed, once we found that endothelium-removed aorta of pregnant groups had greater vasoconstrictor responses than non-pregnant groups. Thirdly, multiple added mechanisms operating to control nitric oxide and other MMPs (MMP-1, MMP-3, MMP-9, MMP-12, and MMP-13) in the range of 3–20 h in disordered pregnancy, such as preeclampsia (a slow-developing condition manifested after weeks of pregnancy), are also needed to be examined in future studies with isoflurane anesthesia in pregnancy [35]. Lastly, changes in the cardiac expression of MMPs and tissue inhibitors of MMPs (TIMPs) suggest that extracellular collagen of the heart is also a target for MMPs in pregnancy [36], and this is worth considering in further investigations with isoflurane.

## Conclusion

This study demonstrated that isoflurane anesthesia at the final stage of pregnancy shows to be reliable in healthy pregnancy because hemodynamic parameters were stable and within normal limits *in vivo*, and fetal weight *ex vivo* is not impaired, and vasoconstrictor responses and MMP-2 activation are not influenced by isoflurane anesthesia. Finally, our data suggest that isoflurane-induced protective effects observed in pregnancy are endothelium-derived nitric oxide dependent.

## Supplementary Material

Supplementary Figures S1-S2

## Data Availability

Authors declare that all the data supporting the results of the present study are included in the article.

## Abbreviations

ACh: acetylcholine
DAP: diastolic arterial pressure
HR: heart rate
MAP: mean arterial pressure
MMP: matrix metalloproteinase
Phe: phenylephrine
SAP: systolic arterial pressure
