# Supplementary material for: Isoflurane increases the activity of the vascular matrix metalloproteinase-2 in non-pregnant rats and increases the nitric oxide metabolites in pregnancy
Source: Biosci Rep. 2024 May 31;44(6):BSR20240192. doi: 10.1042/BSR20240192 (PMC11147811; doi:10.1042/BSR20240192)
Supplement: Supplementary Figures S1-S2 [file BSR-2024-0192_supp.pdf]

Full uncropped and unedited versions of the zymography gels:

Zymography gel 1 of thoracic aorta

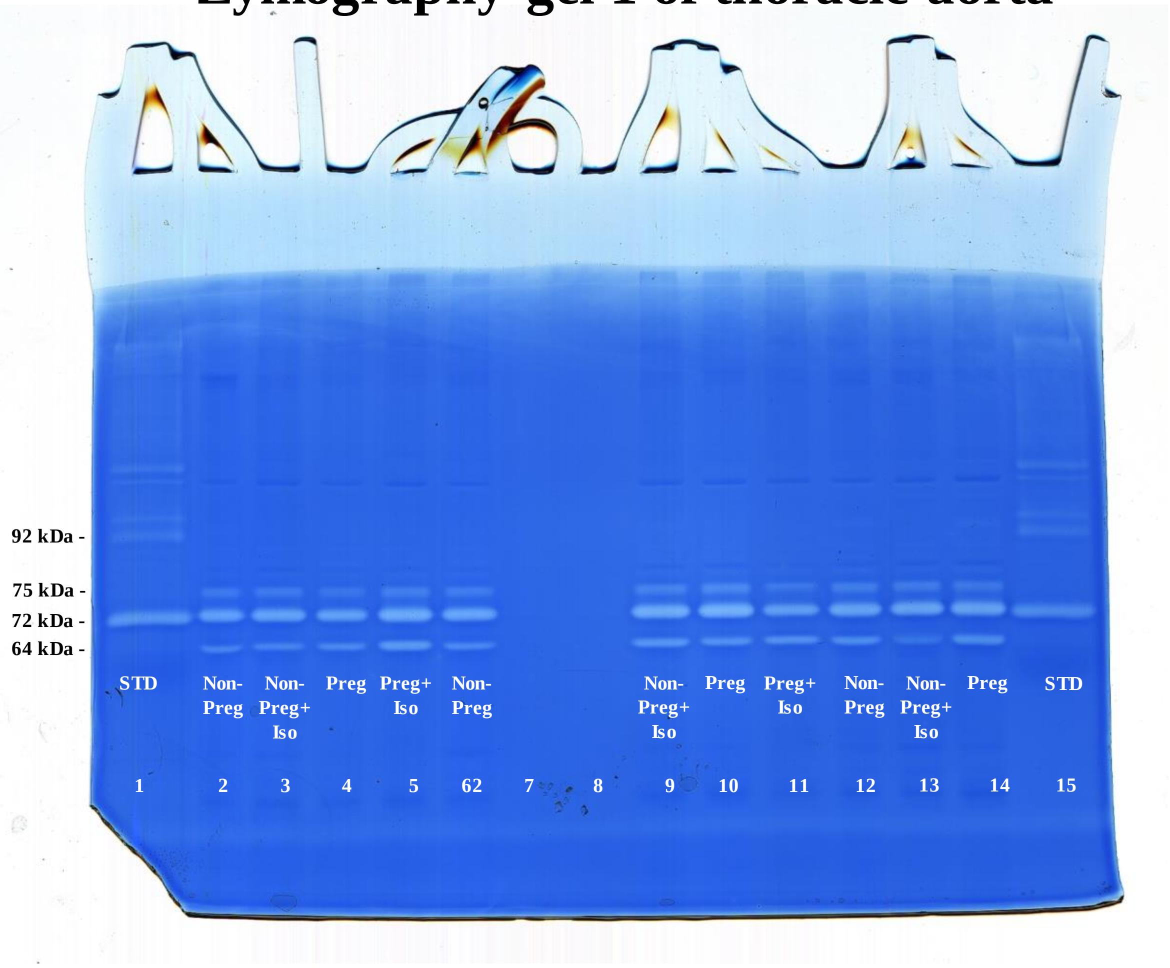

Zymography gel 2 of thoracic aorta

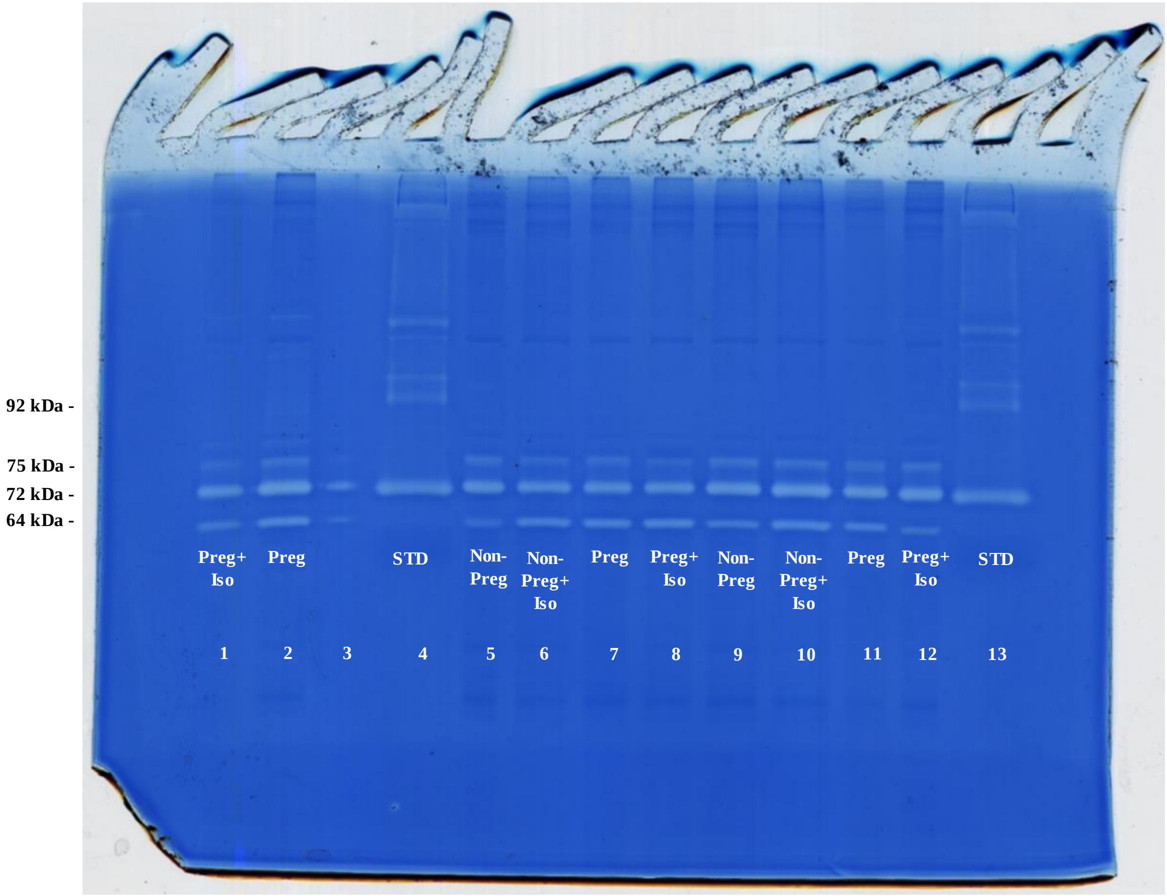

Zymography gel 3 of thoracic aorta

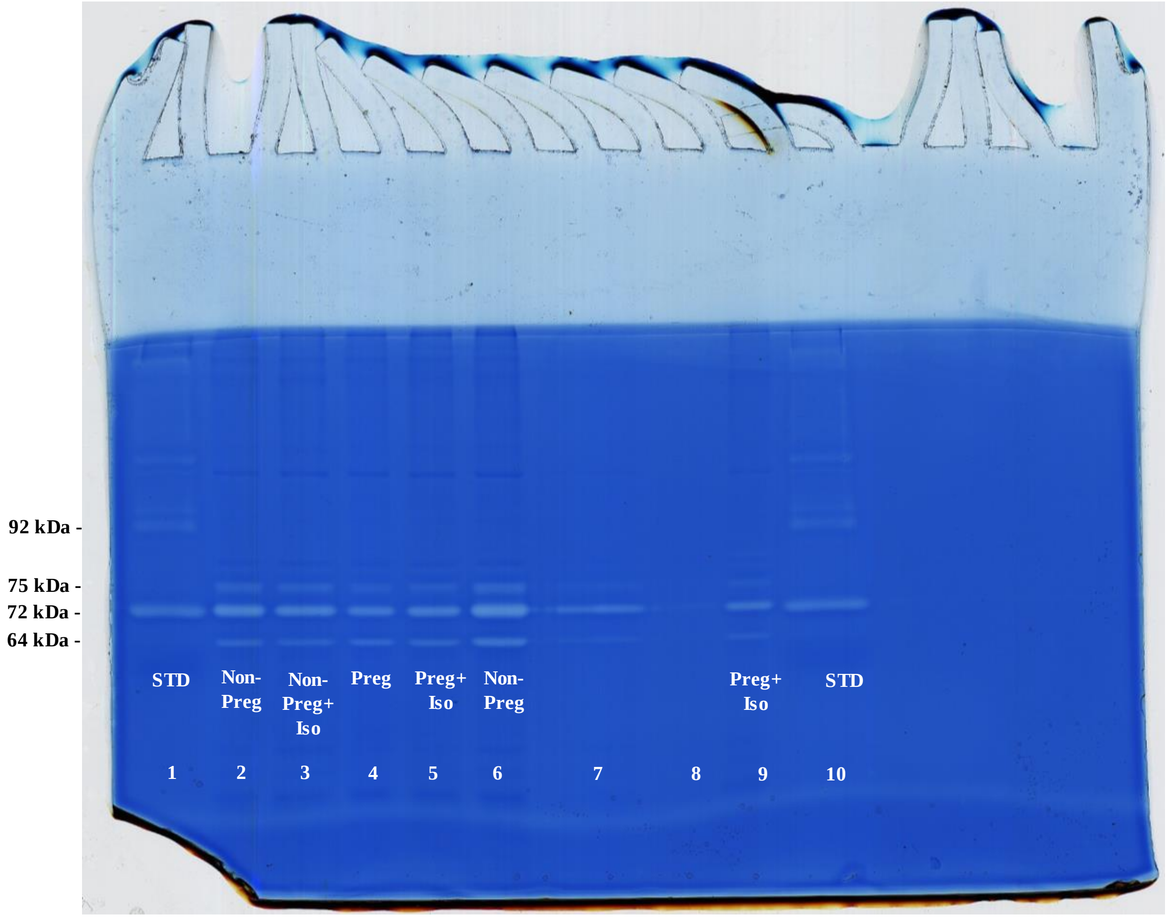

Supplementary Fig S1. Acrylamide gels (1, 2 and 3) stained with Coomassie Brilliant Blue to quantify gelatinolytic activities of 75 kDa MMP-2, 72 kDa MMP-2, and 64 kDa MMP-2 in thoracic aorta from Non-Preg, Non-Preg+Iso, Preg, and Preg+Iso groups. The lanes 2-5 of Gel 3 were used as the representative gel in the figure 3. STD: internal standard.

Zymography gel 1 of plasma

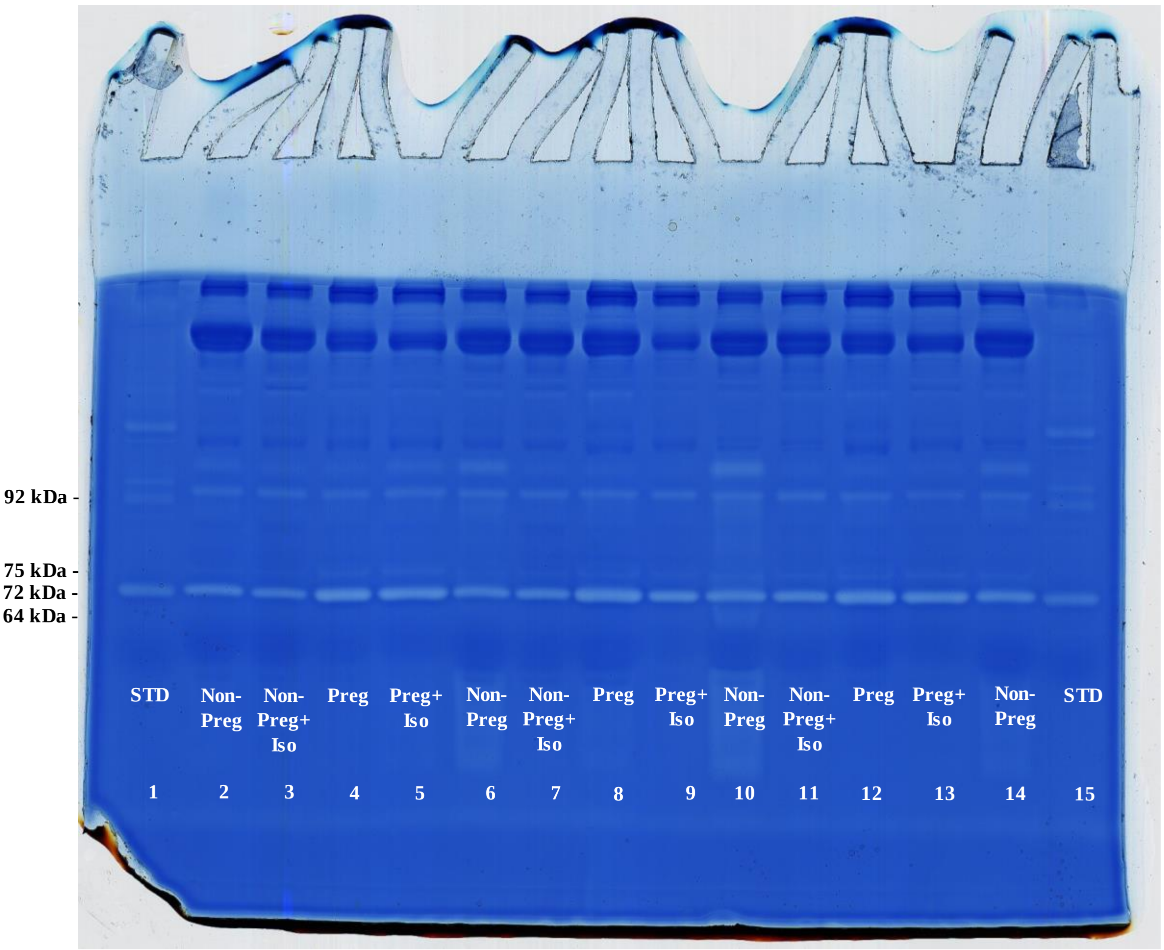

Zymography gel 2 of plasma

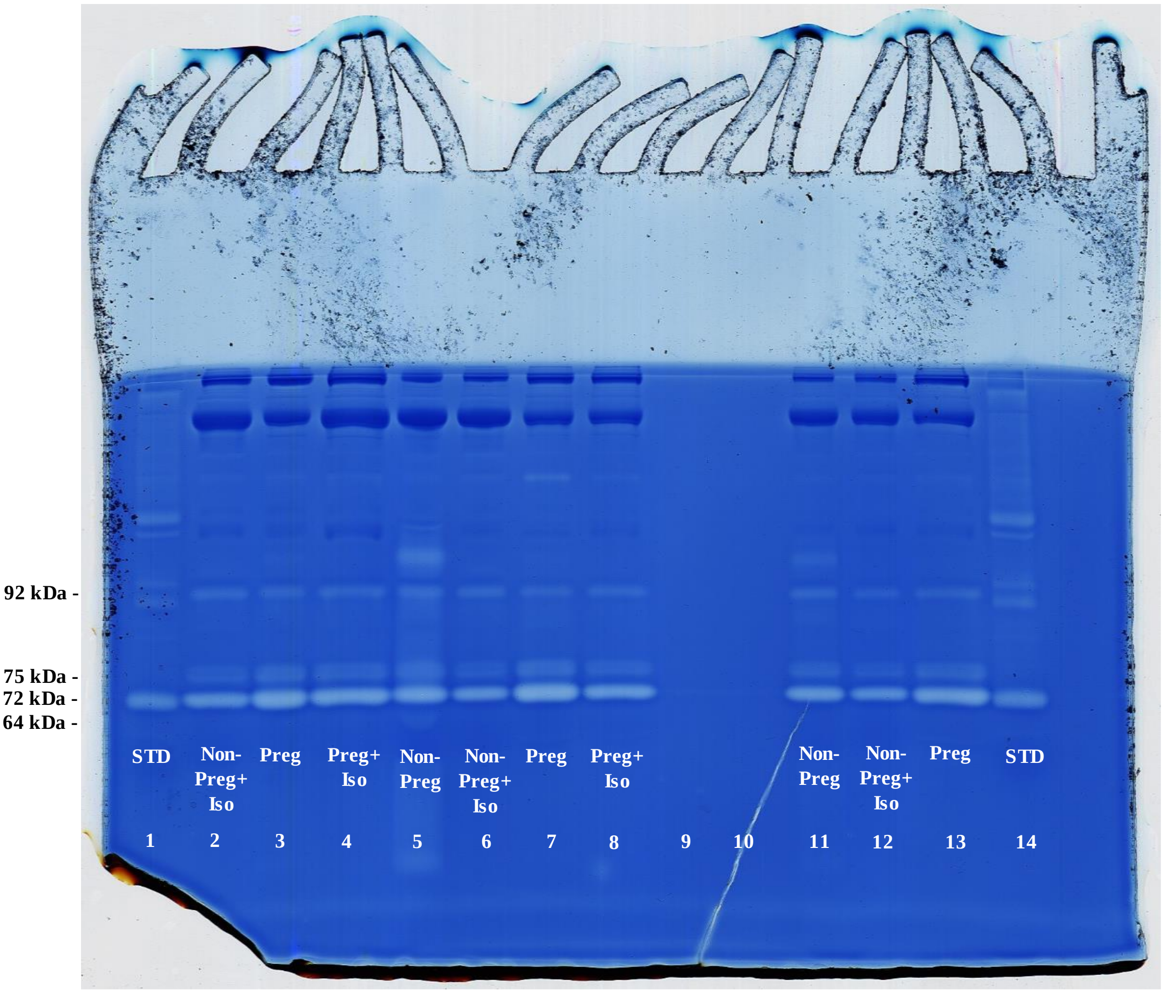

Supplementary Fig S2. Acrylamide gels (1 and 2) stained with Coomassie Brilliant Blue to quantify gelatinolytic activities of 92 kDa MMP-9 and 72 kDa MMP-2 in plasma from Non-Preg, Non-Preg+Iso, Preg, and Preg+Iso groups. The lanes 2-5 of Gel 1 were used as the representative gel in the figure 4. STD: internal standard.
